# Tango1L but not Tango1S, Tali and cTAGE5 is required for export of type II collagen in medaka fish

**DOI:** 10.1247/csf.25001

**Published:** 2025-01-23

**Authors:** Yusuke Yasuda, Tomoka Yoshida, Mahiro Oue, Masaya Sengiku, Tokiro Ishikawa, Shunsuke Saito, Byungseok Jin, Kazutoshi Mori

**Affiliations:** 1 Kyoto University Institute for Advanced Study, Kyoto 606-8501, Japan; 2 Department of Biophysics, Graduate School of Science, Kyoto University, Kyoto 606-8502, Japan

**Keywords:** intracellular transport, COPII vesicles, enlargement, endoplasmic reticulum, Golgi apparatus

## Abstract

Newly synthesized proteins destined for the secretory pathway are folded and assembled in the endoplasmic reticulum (ER) and then transported to the Golgi apparatus via COPII vesicles, which are normally 60–90 nm. COPII vesicles must accordingly be enlarged to accommodate proteins larger than 90 nm, such as long-chain collagen. Key molecules involved in this enlargement are Tango1 and Tango1-like (Tali), which are transmembrane proteins in the ER encoded by the *MIA3* and *MIA2* genes, respectively. Interestingly, two splicing variants are expressed from each of these two genes: Tango1L and Tango1S from the *MIA3* gene, and Tali and cTAGE5 from the *MIA2* gene. Here, we constructed Tango1L-knockout (KO), Tango1S-KO, Tali-KO, and cTAGE5-KO separately in medaka fish, a vertebrate model organism, and characterized them. Results showed that only Tango1L-KO conferred a lethal phenotype to medaka fish. Only Tango1L-KO medaka fish exhibited a shorter tail than wild-type (WT) fish and showed the defects in the export of type II collagen from the ER, contrary to the previous reports analyzing Tango1-KO or Tali-KO mice and the results of knockdown experiments in human cultured cells. Medaka fish may employ a simpler system than mammals for the export of large molecules from the ER.

## Graphical Abstract

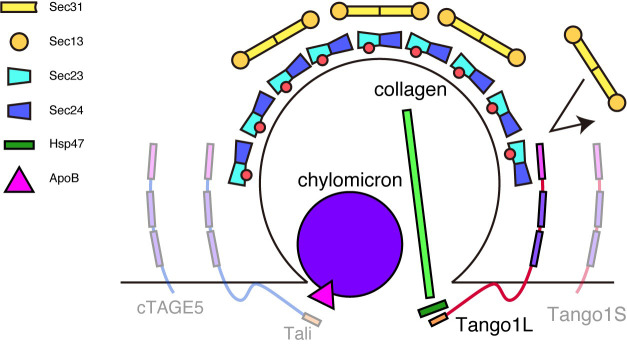

## Introduction

**Fig. 1:**
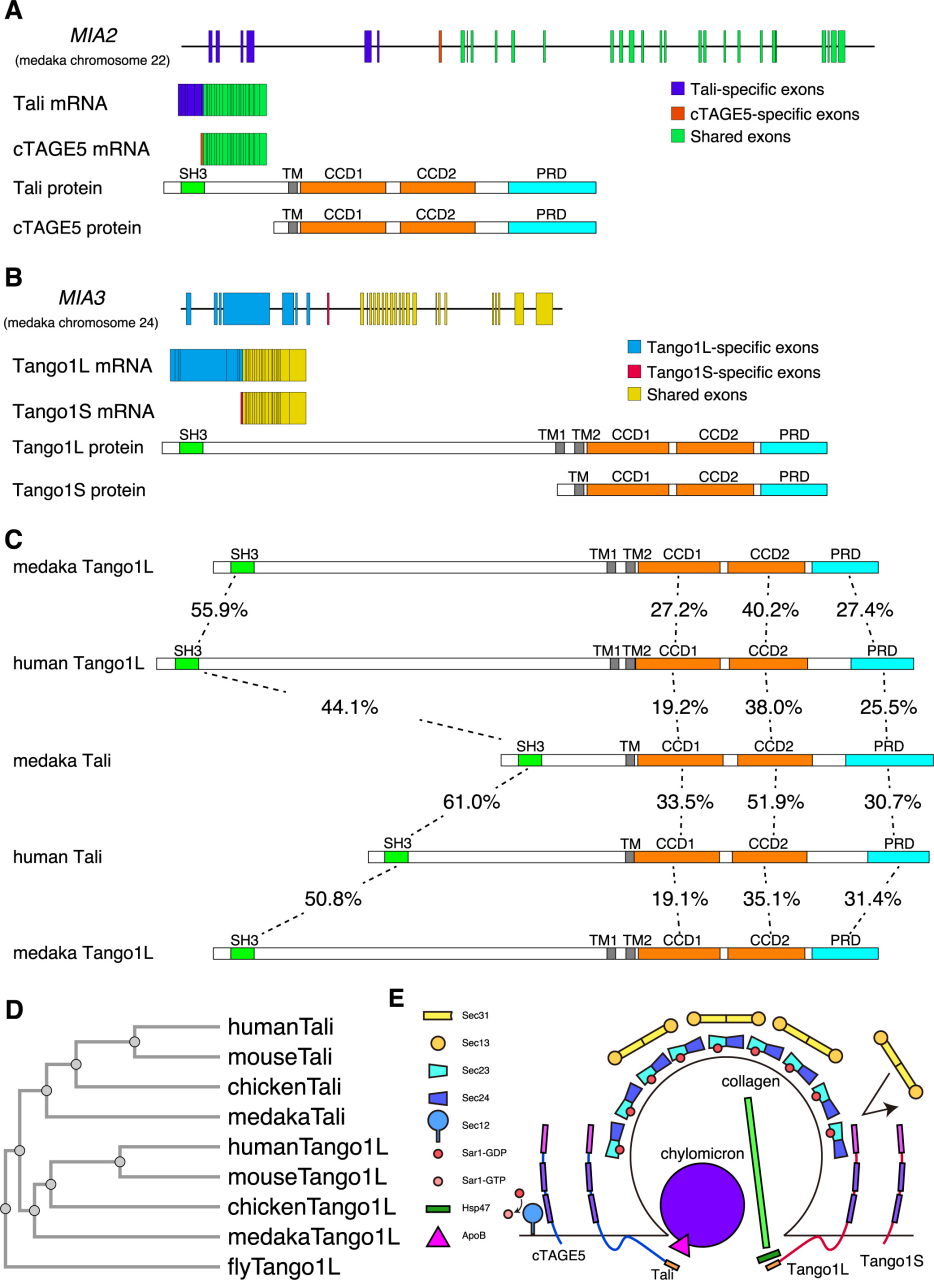
Structures of the *MIA2* and *MIA3* genes as well as comparison of Tali and Tango1L (A) Structures of the *MIA2* gene, Tali and cTAGE5 mRNAs, and Tali and cTAGE5 proteins. (B) Structures of the *MIA3* gene, Tango1L and Tango1S mRNAs, and Tango1L and Tango1S proteins. (C) Sequence identity of domains in Tango1L of medaka and human as well as Tali of medaka and human. (D) Phylogenic tree of Tango1L and Tali. (E) Model of actions of Tango1L, Tango1S, Tali, and cTAGE5.

Newly synthesized proteins destined for the secretory pathway are folded and assembled in the ER with assistance from ER-localized molecular chaperones and folding enzymes, and only correctly folded molecules are allowed to move to the Golgi apparatus (Bukau *et al.*, 2006). This transport from the ER is mediated by COPII vesicles, which are covered by two layers of protein complex, namely an inner coat consisting of Sec23 and Sec24 as well as an outer coat consisting of Sec13 and Sec31, and then depart from the ER exit site (see Fig. 1E). The size of COPII vesicles is normally 60–90 nm (Raote *et al.*, 2023).

However, the cell sometimes needs to transport cargo proteins that are larger than 90 nm. For example, during formation of the notochord, notochord cells are smoothly aligned by synthesizing and secreting large amounts of extracellular matrix proteins, including type VIII collagen (Ishikawa *et al.*, 2017). After secretion of hedgehog to regulate patterning of various surrounding tissues (Stemple, 2005), notochord cells receive the Mib-Jag1-Notch signal and then differentiate into two types of cells: one is large vacuolated structural cells that generate turgor and the other is thin non-vacuolated epithelial cells (sheath cells). As the notochord is a primitive form of cartilage, sheath cells synthesize and secrete type II collagen to form the peri-notochordal basement membrane, which covers the notochord as a sheath. Thanks to turgor generated by vacuolated cells and the sheath formed by sheath cells, the notochord extends smoothly and rigidly and thereby functions as an axial skeleton before the formation of vertebra (Ishikawa *et al.*, 2017; Yamamoto *et al.*, 2010).

Long-chain collagen such as type II collagen is longer than 300 nm and therefore cannot be incorporated into standard COPII vesicles. How such large cargo proteins could be transported was a major question in cell biology (Fromme and Schekman, 2005). In 2009, Vivek Malhotra and colleagues discovered an interesting and important molecule termed transport and Golgi organization (Tango) 1, which is localized in the ER as a transmembrane protein (Saito *et al.*, 2009). Its N-terminal SH3 domain binds to long-chain collagen via Hsp47 in the lumen of the ER, whereas its C-terminal proline-rich domain (PRD) binds to Sec23/Sec24 in the cytosol, which blocks the completion of the formation of the outer coat layer, resulting in the enlargement of COPII vesicles to accommodate long-chain collagen (see Fig. 1E) (Malhotra and Erlmann, 2015).

Following the finding that a truncated version of Tango1 which lacked a majority of the N-terminal region was expressed in cultured cells, the full-length and truncated versions of Tango1 (note that they are splicing variants) were designated as Tango1L and Tango1S, respectively (Maeda *et al.*, 2016; Wilson *et al.*, 2011). Interestingly, cutaneous T-cell lymphoma-associated antigen 5 (cTAGE5) was found to be substantially homologous to Tango1S, and accordingly its full-length version was named Tango1-like (Tali); Tali and cTAGE5 are also splicing variants (Santos *et al.*, 2016). Since then, the roles of Tango1L/Tango1S and Tali/cTAGE5 in the export of long-chain collagen as well as triglyceride-rich chylomicron (~150–500 nm in diameter) have been extensively investigated using cultured cells (Raote *et al.*, 2021; Raote *et al.*, 2023).

At the animal levels, Tango1-knockout (KO) mice were constructed and characterized (Wilson *et al.*, 2011). Because a region containing the N-terminal SH3 domain was deleted, these Tango1-KO mice did not express Tango1L but did express Tango1S. These Tango1L-KO mice showed defects in the secretion of various collagens from chondrocytes, fibroblasts, endothelial cells, and mural cells; exhibited short-limbed dwarfism; and died at birth. On the other hand, Tali/cTAGE5-KO mice were constructed by deleting a cytoplasmic small region inside the shared coiled-coiled domain (CCD) 2, which lead to the loss of functionally important PRD capable of binding to Sec23/Sec24 in both Tali and cTAGE5. These KO mice showed embryonic lethalality (Wang *et al.*, 2016).

In zebrafish (Clark and Link, 2021), as a majority of the N-terminal luminal region was deleted, Tango1-KO zebrafish did not express Tango1L but did express Tango1S, similarly to Tango1L-KO mice. These Tango1L-KO zebrafish were shorter than WT fish during the embryonic period, showed craniofacial defects, and did not survive to adulthood. On the other hand, as a small region inside the shared CCD2 was deleted, Tali/cTAGE5-KO zebrafish expressed truncated Tali and cTAGE5 lacking functionally important PRD capable of binding to Sec23/Sec24, similarly to Tali/cTAGE5-KO mice. Tali/cTAGE5-KO zebrafish appeared to be normal during the embryonic period but became shorter than WT fish at 2 months after hatching, and died by 1 year, although WT and heterozygous fish lived longer than 3 years.

Here, we separately knocked out Tango1L, Tango1S, Tali, and cTAGE5 in medaka fish, a vertebrate model organism, using genome editing and characterized their phenotypes for the first time.

## Results

### Relationship between medaka Tango1 and Tali

Two splicing variants are expressed from the *MIA2* gene (MIA stands for melanoma inhibitory activity member) in medaka fish, namely Tali mRNA and cTAGE5 mRNA. Thus, the *MIA2* locus possesses 6 Tali-specific exons, 1 cTAGE5-specific exon, and 22 shared exons from the 5' end to the 3' end (Fig. 1A). Similarly, two splicing variants are expressed from the *MIA3* gene in medaka fish, namely Tango1L mRNA and Tango1S mRNA. Thus, the *MIA3* locus possesses 6 Tango1L-specific exons, 1 Tango1S-specific exon, and 21 shared exons from the 5' end to the 3' end (Fig. 1B). Both Tali and Tango1L contain SH3, transmembrane (TM) domain, CCD1, CCD2, and PRD, whereas both cTAGE5 and Tango1S lack a majority of the luminal region present in Tali and Tango1L (Fig. 1A, B, E). Sequence comparison shows that human Tango1L is more similar to medaka Tango1L than to medaka Tali, whereas human Tali is more similar to medaka Tali than to medaka Tango1L (Fig. 1C, D).

### Construction and characterization of Tali- and cTAGE5-knockout medaka

**Fig. 2:**
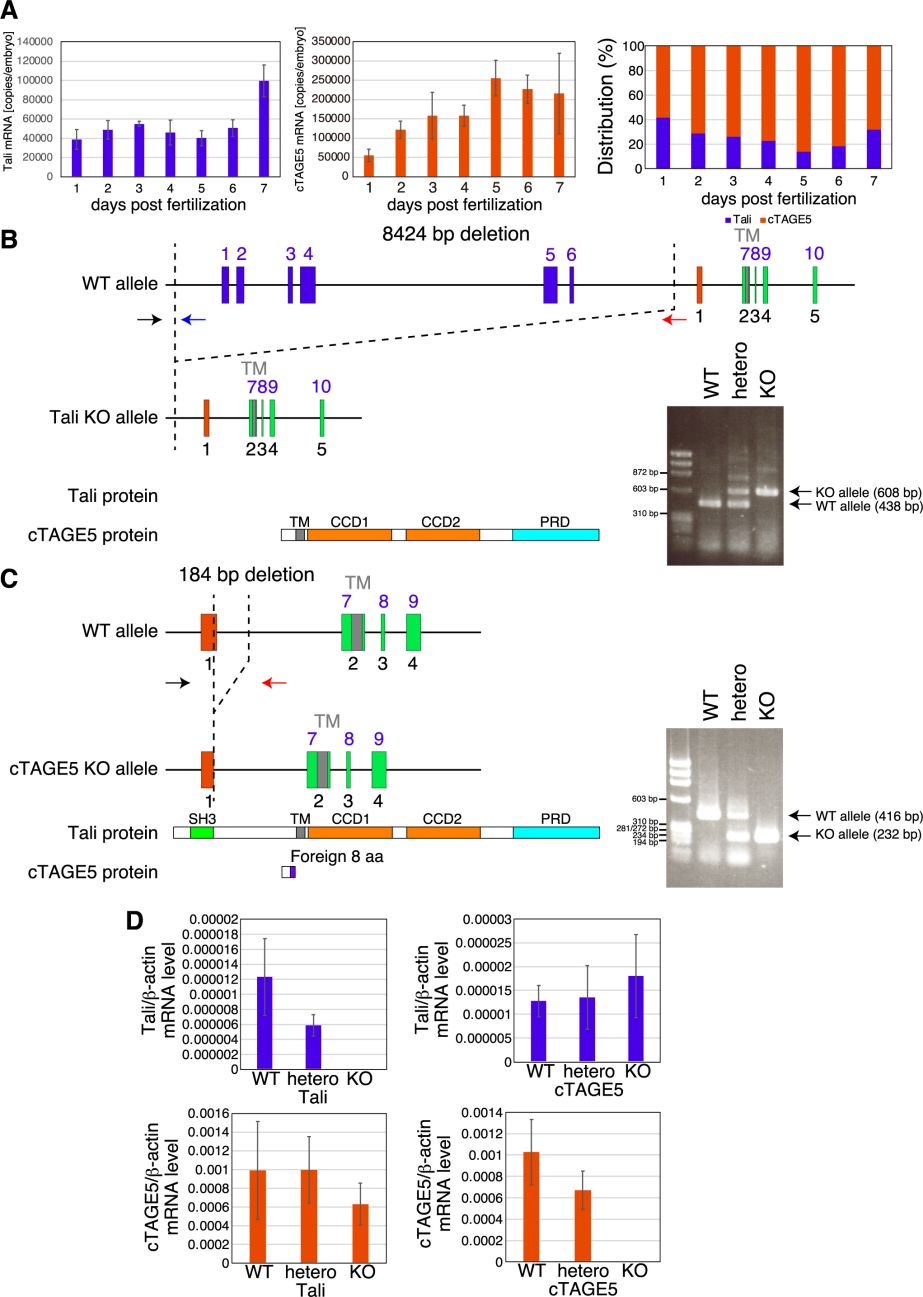
Construction of Tali-KO medaka and cTAGE5-KO medaka (A) Quantitative RT-PCR analysis to determine absolute expression levels of Tali mRNA and cTAGE5 mRNA in WT embryos of 1–7 dpf. (B) Strategy to construct Tali-KO medaka and confirmation by genomic PCR. (C) Strategy to construct cTAGE5-KO medaka and confirmation by genomic PCR. (D) Quantitative RT-PCR analysis to determine expression levels of Tali mRNA and cTAGE5 mRNA relative to β-actin mRNA in WT, hetero, and KO medaka of Tali and cTAGE5 using caudal fin at 7 mph.

Determination of the absolute expression levels of Tali mRNA and cTAGE5 mRNA by quantitative RT-PCR using full-length Tali cDNA amplified by PCR as standard showed that cTAGE5 mRNA was more abundantly expressed than Tali mRNA in embryos from 1 to 7 days post-fertilization (dpf) (Fig. 2A, medaka usually hatch at 7 dpf).

We constructed Tali-KO medaka and cTAGE5-KO medaka using CRISPR/Cas9-mediated genome editing. To construct Tali-KO medaka, the exons 1–6 of the *MIA2* gene were deleted entirely, which was confirmed by genomic PCR (Fig. 2B). This Tali-KO medaka did not express Tali mRNA but did express cTAGE5 mRNA as expected, when examined at 7 months post-hatching (mph) (Fig. 2D; medaka gain reproductive capacity at 2~3 mph). To construct cTAGE5-KO medaka, the cTAGE5-specific exon of the *MIA2* gene was deleted, which was confirmed by genomic PCR (Fig. 2C). This cTAGE5-KO medaka expressed Tali mRNA but did not express cTAGE5 mRNA at 7 mph, as expected (Fig. 2D).

**Fig. 3:**
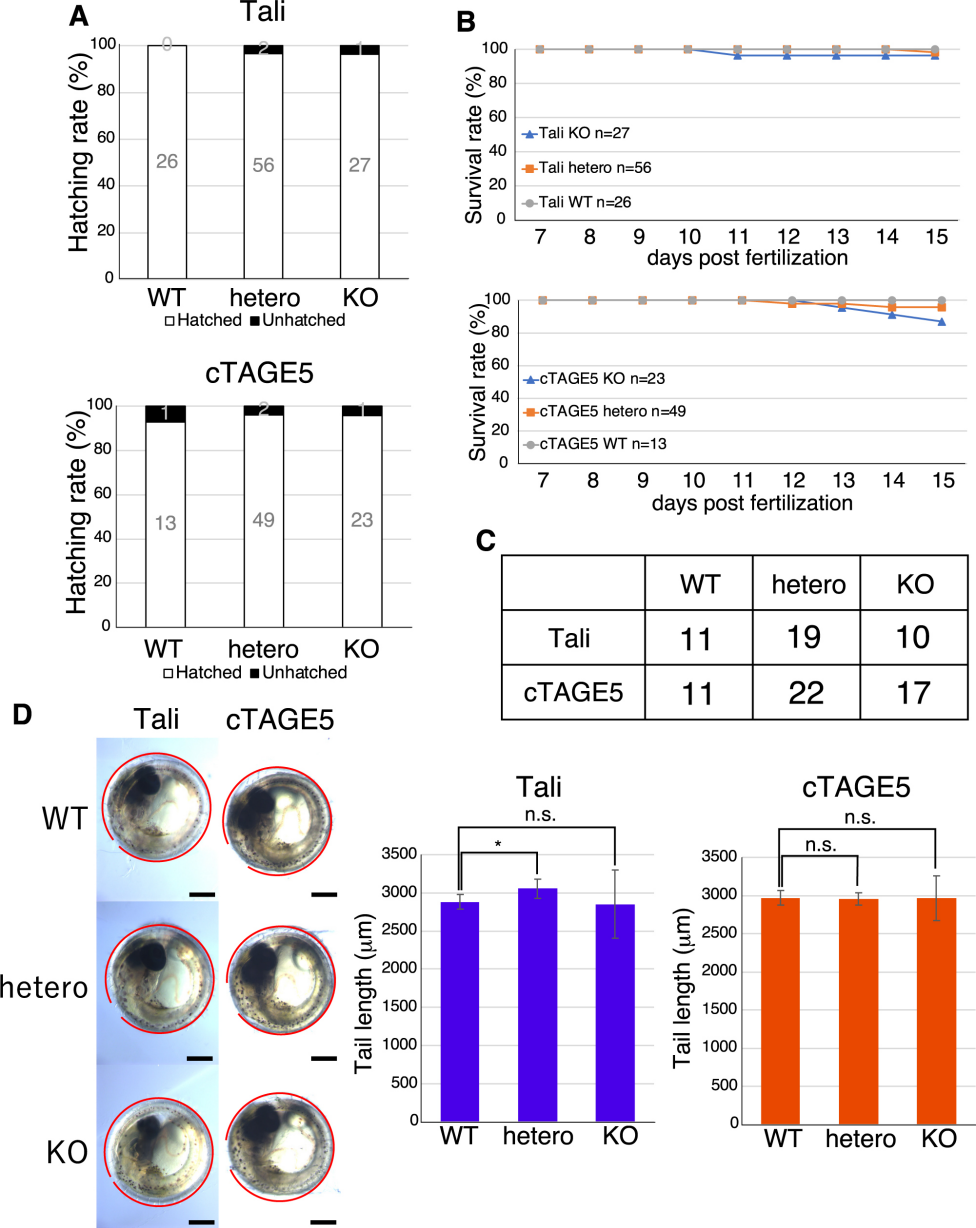
Characterization of Tali-KO medaka and cTAGE5-KO medaka (A) Hatching rate of WT, hetero, and KO medaka of Tali and cTAGE5. (B) Survival rate of WT, hetero, and KO medaka of Tali and cTAGE5 from 7 to 15 dpf. (C) Survival of WT, hetero, and KO medaka of Tali and cTAGE5 at 7 mph. (D) Tail length of WT, hetero, and KO medaka of Tali and cTAGE5. Position of the tail is shown with a red line. Scale bar: 300 μm.

A great majority of Tali-KO medaka and cTAGE5-KO medaka hatched (Fig. 3A), and survived until 15 dpf (Fig. 3B) as well as 2 mph (Fig. 3C). Both Tali-KO medaka and cTAGE5-KO medaka exhibited normal tail length (Fig. 3D).

### Construction and characterization of Tango1L- and Tango1S-knockout medaka

**Fig. 4:**
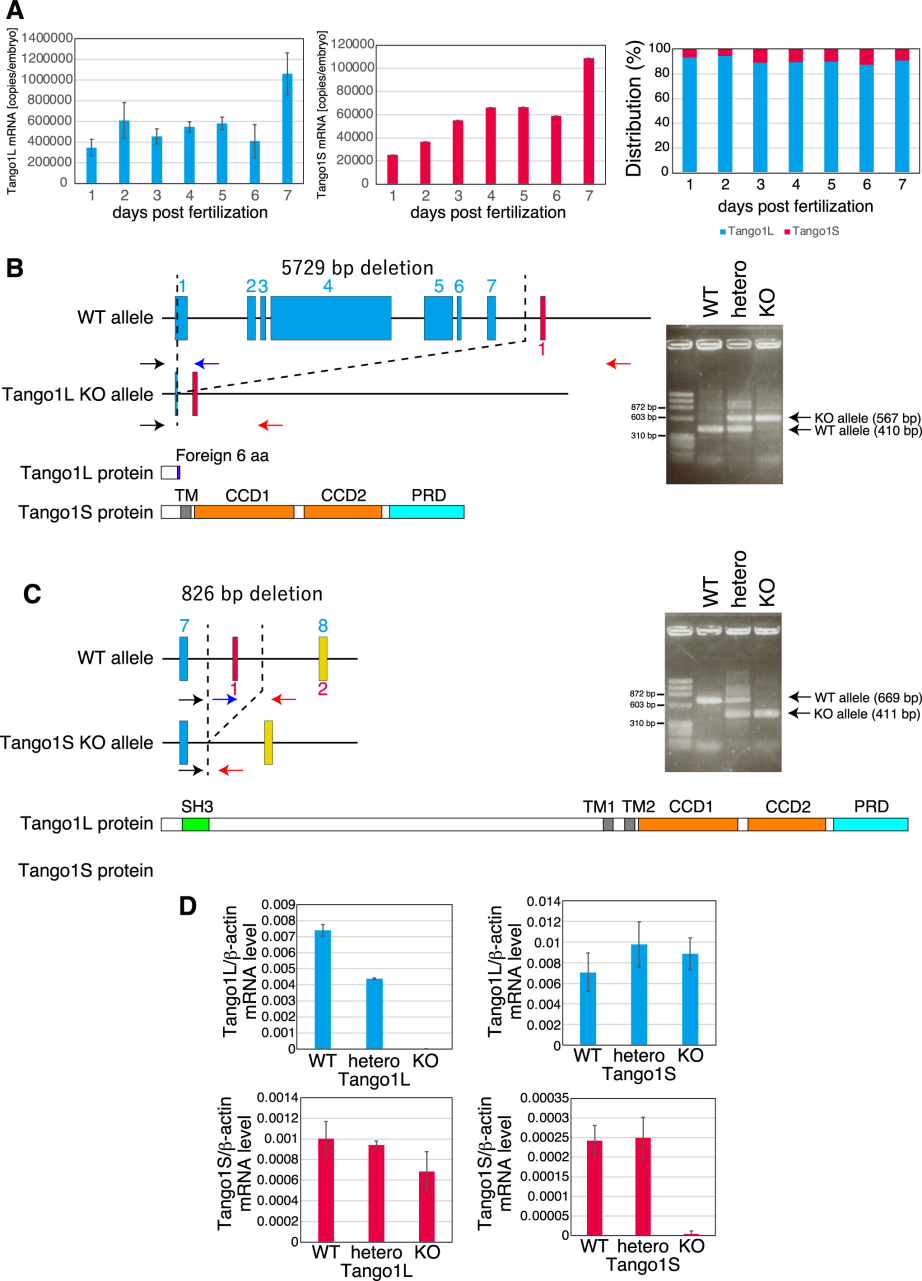
Construction of Tango1L-KO medaka and Tango1S-KO medaka (A) Quantitative RT-PCR analysis to determine absolute expression levels of Tango1L mRNA and Tango1S mRNA in WT embryos of 1–7 dpf. (B) Strategy to construct Tango1L-KO medaka and confirmation by genomic PCR. (C) Strategy to construct Tango1S-KO medaka and confirmation by genomic PCR. (D) Quantitative RT-PCR analysis to determine expression levels of Tango1L mRNA relative β-actin mRNA in WT, hetero, and KO medaka of Tango1L using embryos at 3 dpf (because Tango1L-KO medaka are not present at 7 mph; see Fig. 5C), as well as expression levels of Tango1S mRNA relative to β-actin mRNA in WT, hetero, and KO medaka of Tango1S using caudal fin at 7 mph.

Determination of the absolute expression levels of Tango1L mRNA and Tango1S mRNA by quantitative RT-PCR using a plasmid carrying Tango1 cDNA as standard showed that approximately 90% were Tango1L mRNA in embryos from 1 to 7 dpf (Fig. 4A).

We constructed Tango1L-KO medaka and Tango1S-KO medaka using CRISPR/Cas9-mediated genome editing. To construct Tango1L-KO medaka, the exons 1–7 of the *MIA3* gene were deleted almost entirely, which was confirmed by genomic PCR (Fig. 4B). This Tango1L-KO medaka did not express Tango1L mRNA but did express Tango1S mRNA, as expected (Fig. 4D). To construct Tango1S-KO medaka, the Tango1S-specific exon of the *MIA3* gene was deleted, which was confirmed by genomic PCR (Fig. 4C). This Tango1S-KO medaka expressed Tango1L mRNA but did not express Tango1S mRNA, as expected (Fig. 4D).

**Fig. 5:**
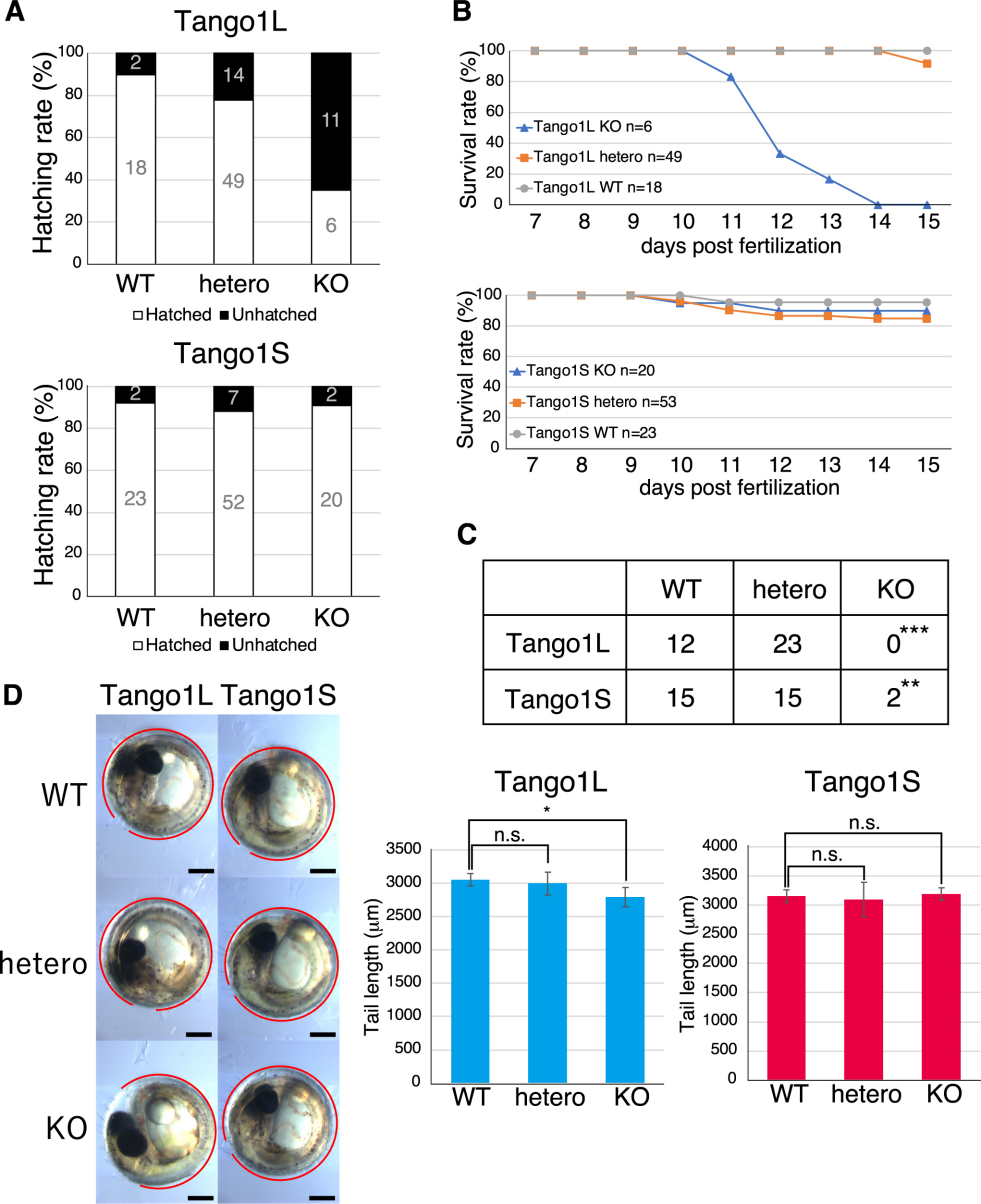
Characterization of Tango1L-KO medaka and Tango1S-KO medaka (A) Hatching rate of WT, hetero, and KO medaka of Tango1L and Tango1S. (B) Survival rate of WT, hetero, and KO medaka of Tango1L and Tango1S from 7 to 15 dpf. (C) Survival of WT, hetero, and KO medaka of Tango1L and Tango1S at 6 mph. (D) Tail length of WT, hetero, and KO medaka of Tango1L and Tango1S. Position of the tail is shown with a red line. Scale bar: 300 μm.

Approximately two-thirds of Tango1L-KO medaka did not hatch (Fig. 5A). Hatched Tango1L-KO medaka started to die from 11 dpf and all died by 14 dpf (Fig. 5B, C). In contrast, a great majority of Tango1S-KO medaka hatched (Fig. 5A) and survived until 15 dpf (Fig. 5B). However, it should be noted that Tango1S-KO medaka survived for 6 mph did not show the Mendelian ratio (Fig. 5C). Tango1L-KO medaka but not Tango1S-KO medaka exhibited a shorter tail than WT fish (Fig. 5D).

### Export of type II collagen

**Fig. 6:**
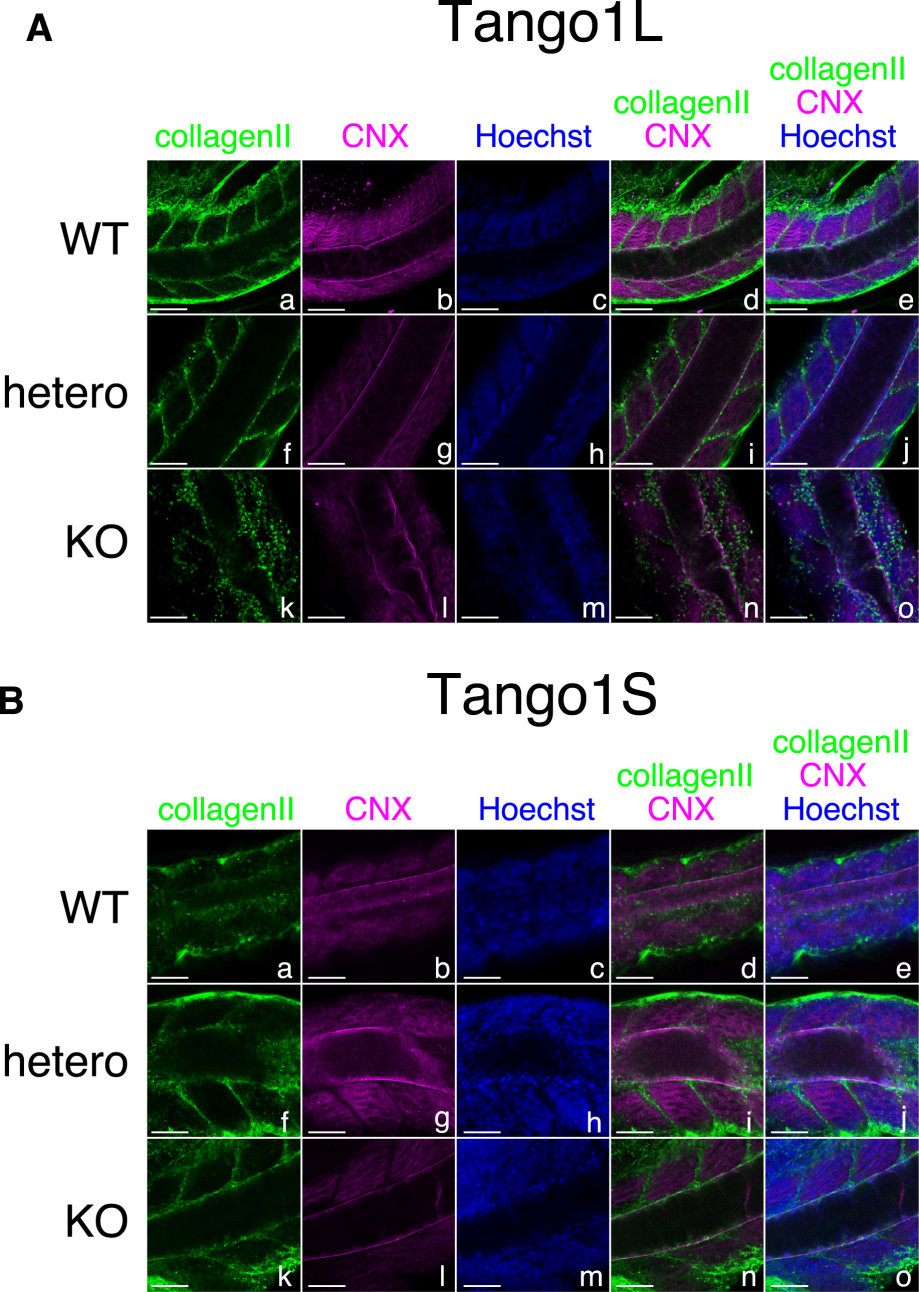
Export of type II collagen in Tango1L-KO medaka and Tango1S-KO medaka (A) (B) WT, hetero, and KO embryos at 3 dpf of Tango1L (A) and Tango1S (B) were analyzed by immunofluorescence using anti-type II collagen and anti-calnexin antibodies. Scale bar: 50 μm.

As only Tango1L-KO medaka exhibited several phenotypes, immunofluorescence analysis of type II collagen was conducted with comparison of calnexin (CNX), an ER marker, in embryos with various genotypes of Tango1L and Tango1S, focusing on the region containing the notochord. Type II collagen was secreted to form the peri-notochordal basement membrane immediately outside CNX-positive ER in Tango1L-WT, Tango1L-hetero, Tango1S-WT, Tango1S-hetero, and Tango1S-KO medaka (Fig. 6A, B). Notably, type II collagen remained punctate and the peri-notochordal basement membrane was not formed in Tango1L-KO medaka (Fig. 6A, panel k).

## Discussion

Two splicing variants are expressed from the *MIA3* gene, namely Tango1L mRNA and Tango1S mRNA. Our present analysis revealed that Tango1L but not Tango1S is required for the export of type II collagen from the ER (Fig. 6). This is in contrast to previous Tango1S knockdown experiments, which showed that Tango1S is also required for the secretion of type VII collagen from the ER in human cultured cells (Maeda *et al.*, 2016). This discrepancy may be due to the difference in their expression levels: Tango1L mRNA is approximately 9-fold more abundant than Tango1S mRNA in medaka fish (Fig. 4A), whereas immunoblotting showed that Tango1S is abundantly expressed in addition to Tango1L in human cultured cells (Maeda *et al.*, 2016). Considering that Tango1L and Tango1S are interchangeable in human cultured cells (Maeda *et al.*, 2016), medaka Tango1L alone would be able to initiate the enlargement of COPII vesicles to accommodate long-chain collagen without making heterodimers with Tango1S. Accordingly, the absence of Tango1L hampered the formation of the peri-notochordal basement membrane, leading to a short tail (Fig. 5D), because turgor generated by vacuolated cells could not be efficiently utilized to extend the notochord smoothly and rigidly.

Nonetheless, given that Tango1S-KO medaka surviving for 2 months after hatching did not show the Mendelian ratio (Fig. 5C), our study might have underestimated the importance of Tango1S. Resolution of this question requires the construction and analysis of Tango1L/Tango1S-double KO medaka. Also, the cause of the death of Tango1L-KO medaka within 14 dpf should be determined, because it cannot be explained solely by short tail. Furthermore, the question of whether the transport defects in Tango1L-KO medaka could be rescued by overexpression of Tango1S, Tali, or cTAGE5 needs to be answered.

Two splicing variants are expressed from the *MIA2* gene, namely Tali mRNA and cTAGE5 mRNA. Our current analysis revealed that neither Tali nor cTAGE5 is required for survival (Fig. 3) and for the export of type II collagen from the ER (data not shown) in medaka fish. This is surprising because it was previously shown that cTAGE5 heterodimerizes with Tango1L and is required for the export of type VII collagen in human cultured cells (Saito *et al.*, 2011). As mentioned above, Tango1L is required for the export of type II collagen in medaka fish, but does not seem to rely on a partner(s) for its functionality in this fish. Biochemical analysis is required to understand the difference between mammalian cells and medaka fish; i.e., the importance of heterodimerization in the export of long-chain collagen from the ER. Further, a conclusive determination of the role of Tali/cTAGE5 in the physiology of medaka fish requires the construction and analysis of Tali/cTAGE5-double KO medaka, as was done in zebrafish (Clark and Link, 2021).

## Materials and Methods

### Statistics

Statistical analysis was conducted using Student’s t-test, with probability expressed as *p<0.05, **p<0.01 and ***p<0.001 for all figures.

### Fish

Medaka southern strain Cab was used as WT fish. Fish were maintained in a recirculating system with a 14:10 hr light:dark cycle at 27.5°C. All experiments were performed in accordance with the guidelines and regulations established by the Animal Research Committee of Kyoto University (approval number: H2819).

### CRISPR/Cas9 method

To synthesize sgRNAs for CRISPR/Cas9-mediated genome editing, a PCR product amplified from DR274 vector (Addgene) using T7-sgRNA-Fw and DR274-Rv primers was purified by phenol chloroform extraction and used as template for T7 RNA polymerase. The sequences of transcribed sgRNAs are shown in Table 1.

Cas9 expression vector was linearized with NotI, purified by phenol-chloroform extraction, and used as template to synthesize capped mRNAs using the Message mMachine SP6Kit (Invitrogen).

Synthesized RNAs were purified by phenol chloroform extraction (for sgRNAs) or RNeasy mini kit (Qiagen) (for Cas9 mRNA) and microinjected into one-cell-stage embryos at a concentration of 1 μg/μl for Cas9 and 25 ng/μl for both upstream and downstream targeting sgRNAs. Injection was performed as described previously (Ishikawa *et al.*, 2011).

### Genotyping

Embryos or hatched fish were suspended in 50 μl of lysis buffer (10 mM NaOH and 0.2 mM EDTA), boiled for 10 min, and then neutralized by the addition of an equal volume of 40 mM Tris-HCl, pH 8.0. DNA fragment containing a part of the *MIA2* or *MIA3* gene was amplified by PCR directly from lysates using the primers shown in Table 1.

### Quantitative RT-PCR

Total RNA was extracted from embryos or caudal fin at the indicated dpf or mph by the acid guanidinium-phenol-chloroform method using Isogen (Nippon Gene). Quantitative RT-PCR analysis was carried out as described previously (Ishikawa *et al.*, 2013) using the SYBR Green method (Applied Biosystems) and a pair of primers (Fw and Rv) whose names and sequences are described in Table 1.

### Microscopy and Tail length measurement

Brightfield microscopic analysis was conducted using Leica M205FA stereomicroscope.

Tail length was determined by analyzing microscopic images using ImageJ (https://imagej.nih.gov/ij/).

### Immunofluorescence

Embryos were fixed in 4% paraformaldehyde in PBS overnight at 4°C, washed with PBS, fixed again with 100% methanol for 15 min at room temperature and more than overnight at –30°C, and then washed with PBST (0.1% Tween20 in PBS). Fixed embryos were permeabilized by incubation with 0.1 mg/ml proteinase K in PBSX (0.1% Triton X-100 in PBS) for 22 min at room temperature and washed with PBSDX (1% DMSO and 0.3% Triton X-100 in PBS) three times. Permeabilized embryos were blocked in blocking solution (2% fetal bovine serum and 2 mg/ml BSA in PBSX) for 4 h at 4°C, reacted with primary antibody shown in Table 1 in blocking solution overnight at 4°C, washed two times with PBSDX, reacted with secondary antibody shown in Table 1 and Hoechst 33342 for 1 h at room temperature, and then washed with PBSDX. Immunostained embryos were analyzed using Zeiss LSM880 microscopy.

## Figures and Tables

**Table 1:** Information of DNA sequences and antibodies

sgRNAs	
Target	Sequence
*ol* Tango1L-up	GGGGUUCGCAUCGAUGGAUGGUUUUAGAGCUAGAAAUAGCAAGUUAAAAUAAGGCUAGUCCGUUAUCAACUUGAAAAAGUGGCACCGAGUCGGUGCUUUU
olTango1L-down	GGCGUGUGCUUUCACGGUGAGUUUUAGAGCUAGAAAUAGCAAGUUAAAAUAAGGCUAGUCCGUUAUCAACUUGAAAAAGUGGCACCGAGUCGGUGCUUUU
*ol* Tango1S-up	GGCGUGUGCUUUCACGGUGAGUUUUAGAGCUAGAAAUAGCAAGUUAAAAUAAGGCUAGUCCGUUAUCAACUUGAAAAAGUGGCACCGAGUCGGUGCUUUU
*ol* Tango1S-down	GGCCUGCUACUAUAGUUCAAGUUUUAGAGCUAGAAAUAGCAAGUUAAAAUAAGGCUAGUCCGUUAUCAACUUGAAAAAGUGGCACCGAGUCGGUGCUUUU
*ol* Tali-up	GGGCUUCUGCGGUUGAAUUGGUUUUAGAGCUAGAAAUAGCAAGUUAAAAUAAGGCUAGUCCGUUAUCAACUUGAAAAAGUGGCACCGAGUCGGUGCUUUU
*ol* Tali-down	GGAUUGGCACCUGUUUCCUCGUUUUAGAGCUAGAAAUAGCAAGUUAAAAUAAGGCUAGUCCGUUAUCAACUUGAAAAAGUGGCACCGAGUCGGUGCUUUU
*ol* cTAGE5-up	GGAUUCGCCGUUAACCCAGCGUUUUAGAGCUAGAAAUAGCAAGUUAAAAUAAGGCUAGUCCGUUAUCAACUUGAAAAAGUGGCACCGAGUCGGUGCUUUU
*ol* cTAGE5-down	GGUUCUCCUGUUCGGCUAACGUUUUAGAGCUAGAAAUAGCAAGUUAAAAUAAGGCUAGUCCGUUAUCAACUUGAAAAAGUGGCACCGAGUCGGUGCUUUU

Primers for genotyping	
Target	Sequence
*ol* Tango1L	GAATGAGGTCGTGCTTTCCAGTCTTCGTG
	CATGTGATCTGGTTCCGCTACAGGTTGCTG
	CTCCCGTATCAGGATCAACGGAAGGGAAC
*ol* Tango1S	CATCACGTCTCTCATCAAACGTTGTCAGTG
	CCATCCTATTTAGCTTGATTGGTTTCAGGC
*ol* Tali	GGTGTCTTGTCCTAACTCCACCGGAAGCAG
	GTGACCGTCAAGCATGTACCACAACACTTC
	CAACGTTGCTCGTAACCTGATTGCAGAAC
*ol* cTAGE5	GTTAACCCAGCTGGCAGCTCTACTTTCTAC
	ATTCAGCATAAGAAACAGCTAACTACTAAC

Primers for qRT-PCR	
Target	Sequence
*ol* Tango1L-Fw	CCTGAGCTTGAGCCTTCATT
*ol* Tango1L-Rv	TCCCACTCCTTCCTCTTGTT
*ol* Tango1S-Fw	GTAGCGGAACCAGATCACAT
*ol* Tango1S-Rv	CGGCTTCCACTCTTCTGGTA
*ol* Tali-Fw	GGAGATTCTGAGTGTGAAAGCCTAATGA
*ol* Tali-Rv	CTTAGAAAACGGCAGTCGTTACTCTTGT
*ol* cTAGE5-Fw	ACCCGCGGGCCCACCAGGAAGAGCAGGCACAGACC
*ol* cTAGE5-Rv	ATTACTTGCGGCCGCTTCTGCTGTTTCCAGGTTCCTCTG

Antibody		
Immunogen	Source	Identifier
Anti-Collagen II antibody	abcam	ab185430
Anti-Calnexin antibody	Enzo Life Sciences	ADI-SPA-865-F
anti-Mouse IgG (H + L) Cross-Adsorbed Secondary Antibody, Alexa Fluor™ 488	Thermo Fisher Scientific	A-11017
anti-Rabbit IgG (H + L) Cross-Adsorbed Secondary Antibody, Alexa Fluor™ 568	Thermo Fisher Scientific	A-11011

## Abbreviations

CCD: coiled-coiled domain
dpf: day post-fertilization
dph: day post-hatching
mph: month post-hatching
ER: endoplasmic reticulum
KO: knockout
PRD: proline-rich domain
TM: transmembrane
WT: wild-type
